# Coexistence of Post-essential Thrombocythemia Myelofibrosis With Monoclonal Gammopathy of Undetermined Significance: A Case Report

**DOI:** 10.7759/cureus.29948

**Published:** 2022-10-05

**Authors:** Sarvani Reddy Allam, Mridula Sree Naagendran, Jaison Lawrence Alexander Santhi, Ratesh Khillan

**Affiliations:** 1 Medicine, Katuri Medical College & Hospital, Guntur, IND; 2 Medicine, PSG Institute of Medical Sciences & Research, Coimbatore, IND; 3 Medicine, Government Sivagangai Medical College & Hospital, Pudukkottai, IND; 4 Hematology/Oncology, Kingsbrook Jewish Medical Center, New York City, USA

**Keywords:** hematology-oncology, bone marrow, mgus, thrombocythemia, myelofibrosis

## Abstract

Synchronous dual haematological neoplasms are always challenging in both diagnosis and treatment. Here, we report the case of a 73-year-old African American female patient with the coexistence of post-essential thrombocythemia myelofibrosis with MPL positive mutation and monoclonal gammopathy of undetermined significance (MGUS). Our management plan focuses on her anaemia and regular monitoring for observing MGUS transforming into an aggressive multiple myeloma.

## Introduction

According to the 2016 World Health Organization (WHO) classification, myeloproliferative neoplasms (MPN) can be divided into seven categories: chronic myeloid leukaemia, chronic neutrophilic leukaemia, polycythemia vera (PV), primary myelofibrosis (PMF), essential thrombocythemia (ET), chronic eosinophilic leukaemia-not otherwise specified and MPN, and unclassifiable (MPN-U) [1]. Primary myelofibrosis is characterized by clonal proliferation of hematopoietic stem cells and is associated with debilitating symptoms due to the combined effects of increased proinflammatory cytokines and severe splenomegaly [2]. PMF can be prefibrotic (pre-PMF) or overt based on morphological, clinical, and genetic features. Both PMF and post-ET/post-PV(polycythemia vera) MF have a negative impact on patients' lives and can escalate to the blast phase [3]. PMF is frequently accompanied by mutations of genes such as JAK2, CALR, or MPL. The diagnosis mainly relies on bone marrow analysis as these mutations are expected in almost 90% of patients and serve as a supporting rather than a diagnostic marker for the disease [4].

Monoclonal gammopathy of undetermined significance (MGUS) is an asymptomatic premalignant plasma cell disorder that typically affects 3% of people over 50 and has a 1% likelihood of progressing to malignancy. MGUS is characterized by serum M-protein less than 3 g/dl or 30mg/dl, clonal plasma cell count of less than 10% in bone marrow, and absence of signs of end-organ damage (calcium elevation, renal insufficiency, anaemia, and bone abnormalities (CRAB) symptoms, which can indicate plasma cell myeloma) [5]. To the best of our knowledge, no clear association between MPN and MGUS has been identified in the literature so far [6].

## Case presentation

We report the case of a 73-year-old African American female patient with a medical background of essential hypertension, type 2 diabetes mellitus, dyslipidemia, latent tuberculosis, and Alzheimer’s disease, who was referred to Hematology-Oncology due to the presence of unexplained anaemia for the past five years. On a clear search through history, she was found to have a history of essential thrombocytosis for which she had been treated with hydroxyurea but later discontinued due to worsening anaemia. She denied any constitutional symptoms, sweating, weight loss, itching, and nocturnal fever. Clinical examination was insignificant without organomegaly or lymphadenopathy. Her laboratory results are shown in Table 1. Serum protein electrophoresis showed normal albumin. A monoclonal band of about 1.0 g/L was detected with hypergammaglobulinemia. Serum Kappa to Lambda free light chain ratio was high at 4.41.

**Table 1:** Laboratory results AST: aspartate transaminase; ANC: absolute neutrophil count; ALT: alanine transaminase

Test name	Result	Normal values
Hematology		
Hemoglobin, g/dL	9.9	13–17
Platelets, ×10^3/μL	810	150–400
White blood cell, ×10^3/μL	5.3	4–10
ANC, ×10^3/µL	3.1	2–7
Lymphocytes, ×10^3/μL	1.8	1–3
Monocytes, ×10^3/μL	0.4	0.2–1
Eosinophils, ×10^3/μL	0	0–0.5
Chemistry		
Creatinine, mg/dl	0.9	0.6-1.2
Total protein, g/dl	8	6.1-8.1
Total bilirubin, mg/dl	0.7	0.2-1.2
ALT, U/L	27	0–40
AST, U/L	27	0–37
Corrected calcium, mg/dl	9.5	8.6-10.5
Albumin, g/dl	4.2	3.5-5.7
M band in serum, g/L	1	
Serum light chain kappa, mg/L	72.3	3.3–19.4
Serum light chain lambda, mg/L	16.4	5.7–26.3
Kappa/lambda	4.41	0.26-1.65

Peripheral blood showed thrombocythemia and anaemia with occasional giant and large platelets along with teardrop cells and anisocytosis. Bone marrow biopsy was mildly hypercellular for age with maturing trilineage hematopoiesis (30% cellularity) and myeloid hyperplasia. The myeloid-to-erythroid (M:E) ratio ratio was within the normal range (3:1). There was no increase in mononuclear blast-like cells(less than 5%), confirmed by CD34 immunohistochemical staining. Megakaryocytes have increased in number and exhibit prominent cytologic atypia. The cytologic features of megakaryocytes were typical for marrow fibrosis with bizarre hyperchromatic forms and nuclei with immature features. Mild to focally moderate increases in reticulin fibres (Grade 2 of 3) were noted throughout most sections with a loose reticulin network with many intersections, especially in perivascular areas. No coarse fibres were noted. On differential count, plasma cells were slightly increased at 2%.

Molecular testing was positive for missense mutation in Exon 10 of the MPL gene and negative for insertion/deletion mutation within exon 9 of the CALR gene, JAK2 V617F missense mutation, and BCR-ABL1 gene fusion by RT-PCR.

Based on molecular and morphological findings, it was identified as a case of MPN with features consistent with the fibrotic phase of myelofibrosis with MPL positive mutation. The M-spike on serum electrophoresis and minimally increased plasma cells with kappa predominance and absence of hypercalcemia and renal failure fit the diagnostic criteria favouring a plasma cell disorder, MGUS. After a complete workup, the patient was diagnosed with a case of post-ET myelofibrosis with concomitant MGUS. 

Our management strategy centred on her presenting complaint (long-lasting anaemia) and regular monitoring of MGUS transforming into multiple myeloma. However, due to patients' concerns (denial of bone marrow biopsy), we are not able to proceed with future bone marrow biopsies required to monitor the progression of post-ET fibrosis.

## Discussion

There has been an increasing trend of diagnosis of coexistent dual hematologic malignancies. Usually, the symptoms of secondary malignancies are obscured by the symptoms of primary malignancies. It is suggested that the coexistence of dual hematologic malignancy may be due to defective immunity and increased susceptibility. Management of such coexisting malignancies is often tough [7]. A minimal number of articles report dual hematologic malignancies in the literature.

Reporting of concomitant MPN with MGUS in the literature is very scarce. While earlier studies denied the correlation between the two diseases, recent publications suggest a significant association between the two diseases [8]. The exact reason for the occurrence of MGUS in MPN is still unknown. It is hypothesized that the origin of MGUS in MPN may be due to the elevated release of cytokines due to inflammation leading to the proliferation of existing monoclonal plasma cells. There is also speculation that the relationship may be due to a common hematopoietic precursor [6]. A retrospective chart review by Kotchetkov et al., which showed the prevalence of synchronous dual hematologic malignancies to be 1.51% in 3036 patients, suggests that they are not uncommon and should always be evaluated for in the presence of out-of-ordinary findings [9].

Our patient presented with thrombocytosis and anaemia and tested positive for a missense mutation in Exon 10 of the MPL gene. Bone marrow biopsy morphological findings suggest the fibrotic phase of post-ET myelofibrosis. The M-spike in serum protein electrophoresis with kappa predominance, normal renal parameters, and normal calcium favours the diagnosis of MGUS. It is reported that the best management strategy for patients with MPN-MGUS is to treat MPN and to monitor for the progression of MGUS into multiple myeloma. The treatment should be mainly directed towards multiple myeloma in case MGUS progresses into multiple myeloma. Alkylating agent melphalan has been reported to treat coexistent MPN and multiple myeloma [10].

It is crucial to distinguish pre-PMF from ET since pre-PMF has been shown to have a higher probability of developing into overt PMF than ET, and the 2016 WHO diagnostic criteria are shown in Table 2 [1].

**Table 2:** WHO diagnostic criteria (2016) for pre-primary myelofibrosis and essential thrombocythemia Pre-PMF: pre-primary myelofibrosis; CML: chronic myelogenous leukemia; ET: essential thrombocythemia;  PV: polycythemia vera; MDS: myelodysplastic syndrome; LDH: lactate dehydrogenase

Pre-PMF requires all three major criteria and at least one minor criterion	ET requires all four major criteria (or) first three major criteria + one minor criterion
MAJOR CRITERIA	MAJOR CRITERIA
	Platelet count ≥ 450 × 10^9^/L
Bone marrow biopsy showing megakaryocytic proliferation with atypia, reticulin fibrosis not more than grade 1 and is accompanied by increase in bone marrow cellularity (age-adjusted), granulopoiesis and often decreased erythroid proliferation.	Bone marrow biopsy showing significant megakaryocyte lineage proliferation with increased numbers of enlarged, mature megakaryocytes having hyperlobulated nuclei, rarely with reticulin fibrosis (Grade-1). No significant left shift of neutrophil granulopoiesis or erythropoiesis.
WHO criteria for BCR-ABL1 + CML, ET, PV, MDS, or other myeloid neoplasms are not met.	WHO criteria for BCR-ABL1 + CML, PMF, PV, MDS, or other myeloid neoplasms are not met.
Presence of CALR, JAK2 or MPL mutations (or) presence of another clonal mutation, if above mutations are absent (or) absence of minor bone marrow reticulin fibrosis reactive to a secondary condition including infection, autoimmune disease, malignancy.	Presence of CALR, JAK2 or MPL mutation.
MINOR CRITERIA	MINOR CRITERIA
Anaemia not due to a comorbid condition, leucocyte count: ≥ 11 × 10^9^/L, palpable splenomegaly, elevated LDH.	Clonal marker presence (or) reactive thrombocytosis absence.

## Conclusions

More extensive studies are required to investigate the exact clinical and biological relationship between MPN and MGUS, the effect of co-morbidities, and pre-existing blood disorders on the prognosis of patients with synchronous dual hematologic malignancies, and the best course of the treatment plan for such coexisting hematologic malignancies.
